# A “Prime and Expand” strategy using the multifunctional fusion proteins to generate memory-like NK cells for cell therapy

**DOI:** 10.1007/s00262-024-03765-8

**Published:** 2024-07-03

**Authors:** Niraj Shrestha, Michael J. Dee, Pallavi Chaturvedi, Gilles M. Leclerc, Mary Mathyer, Celeste Dufour, Laura Arthur, Michelle Becker-Hapak, Mark Foster, Ethan McClain, Natalia Valderrama Pena, Karen Kage, Xiaoyun Zhu, Varghese George, Bai Liu, Jack Egan, Christian Echeverri, Meng Wang, Lijing You, Lin Kong, Liying Li, Melissa M. Berrien-Elliott, Matthew L. Cooper, Todd A. Fehniger, Peter R. Rhode, Hing C. Wong

**Affiliations:** 1 HCW Biologics Inc., 2929 N. Commerce Parkway, Miramar, FL 33025 USA; 2 Wugen, Inc., Saint Louis, MO 63108 USA; 3 Division of Oncology, Department of Medicine, Washington University School of Medicine, Saint Louis, MO 63110 USA

**Keywords:** NK cells, Prime and Expand, Adoptive cell therapy, Cytokine-induced memory like NK cells, IL-7, IL-12, IL-15, IL-18, IL-21

## Abstract

Adoptive cellular therapy (ACT) using memory-like (ML) natural killer (NK) cells, generated through overnight ex vivo activation with IL-12, IL-15, and IL-18, has shown promise for treating hematologic malignancies. We recently reported that a multifunctional fusion molecule, HCW9201, comprising IL-12, IL-15, and IL-18 domains could replace individual cytokines for priming human ML NK cell programming (“Prime” step). However, this approach does not include ex vivo expansion, thereby limiting the ability to test different doses and schedules. Here, we report the design and generation of a multifunctional fusion molecule, HCW9206, consisting of human IL-7, IL-15, and IL-21 cytokines. We observed > 300-fold expansion for HCW9201-primed human NK cells cultured for 14 days with HCW9206 and HCW9101, an IgG1 antibody, recognizing the scaffold domain of HCW9206 (“Expand” step). This expansion was dependent on both HCW9206 cytokines and interactions of the IgG1 mAb with CD16 receptors on NK cells. The resulting “Prime and Expand” ML NK cells exhibited elevated metabolic capacity, stable epigenetic *IFNG* promoter demethylation, enhanced antitumor activity in vitro and in vivo, and superior persistence in NSG mice. Thus, the “Prime and Expand” strategy represents a simple feeder cell-free approach to streamline manufacturing of clinical-grade ML NK cells to support multidose and off-the-shelf ACT.

**Supplementary Information:**

The online version contains supplementary material available at 10.1007/s00262-024-03765-8.

## Introduction

There is considerable interest in the development of adoptive cell therapy (ACT) using innate natural killer (NK) cells for patients with hematological cancers based on studies showing that hematopoietic cell transplantation (HCT) containing alloreactive NK cells for acute myeloid leukemia (AML) lowered relapse rates and improved survival [1–3]. Early-phase clinical studies with adoptive blood NK cell transfer also showed leukemia clearance and complete responses in poor prognosis AML patients with no treatment-related graft versus host disease or other major toxicities commonly associated with HCT or T cell-based therapies [4–6]. These studies provided proof-of-principle that NK cell transfer is safe and can be utilized as a cellular immunotherapy, leading to further evaluation of multiple different sources and types of NK cell-based ACT strategies in clinical trials [7, 8].

Unlike T cells, NK cells lack antigen specific receptors and instead recognize targets via the integration of signals from germline DNA encoded activating (that recognize stress-induced ligands) and inhibitory signaling receptors (that bind to MHC class I and class I-like ligands) [9]. This includes specialized activating receptors such as CD16/FcγRIIIa that bind the Fc region of antibodies and mediate antibody-dependent cell-mediated cytotoxicity (ADCC) [10, 11]. NK cells also express a variety of cytokine receptors that tune their activation state (e.g., IL-2, IL-15, IL-12, IL-18) or suppress NK cell function (e.g., TGF-β) [12]. NK cells protect the host from diseased cells through two main functions: direct killing and inter-immune cell communication. Specifically, NK cells kill tumor and virus-infected cells through directed release of cytotoxic granules that contain perforin and granzymes, or engagement of death receptors (e.g., TRAIL and Fas ligand). NK cells also communicate via effector and immunoregulatory cytokines (e.g., IFN-γ) and chemokines that modulate recruitment and activity of other immune cells [13]. However, conventional NK (cNK) cells exhibit modest antitumor activity, and current studies focus on overcoming the challenges of imperfect target recognition, inhibition via constitutive and induced checkpoints, suppressive tumor microenvironments, NK-cell-dysfunction in cancer patients, and limited functional in vivo persistence when used as ACT. Many of these challenges may be overcome by differentiating cNK cells into memory-like (ML) NK cells utilizing combined cytokine receptor activation.

Several forms of innate NK cell memory have been described that differ in their molecular mechanisms and immunobiology based on the memory-initiating stimulus, which include hapten activation, viral infection (especially CMV), and combined activation of cytokine receptors (IL-12, IL-15, and IL-18) [14]. Of these, cytokine-induced memory-like (CIML or ML) NK cells have been extensively characterized in preclinical and clinical studies [15]. ML NK cells have improved target recognition via increased activating receptors and have enhanced function when triggered via CD16/FcγRIIIa to perform ADCC [16, 17]. Further, inhibition via KIR receptors is blunted [18], but ML NK cells retain NKG2A as a potent inhibitory checkpoint [19]. Additionally, increased cytotoxic effector molecules, enhanced production of cytokines and chemokines, and improved metabolic fitness all contribute to improved antitumor response of ML NK cells in vitro and in vivo [17]. In preclinical studies, ML differentiation synergized with chimeric antigen receptor (CAR) engineering to enhance NK cell antitumor responses [20]. These collective properties led to translation in a first-in-human phase 1 clinical trial using a single dose of non-engineered ML NK cell following lymphodepleting chemotherapy to treat patients with relapsed or refractory (rel/ref) AML. This trial revealed that transferred ML NK cells were safe, expanded and persisted in vivo, and led to composite complete remissions in 47% of patients [19]. Subsequent studies using immune compatible ML NK cells to augment HCT or treat post-HCT relapse confirmed their safety, identified in vivo expansion and persistence for > 3 months, and resulted in remissions [21, 22]. Thus, ML NK cells are a promising cellular therapy platform to treat a variety of cancers, but full exploration of dose and schedule requires an ex vivo expansion approach, which retains key aspects of ML NK cell immunobiology. Modern approaches to improve the activity of NK cell therapy utilize multiple sources of NK cells (blood, cord blood, hematopoietic stem cells, progenitor cells, and induced pluripotent stem cells), and include CAR engineering, blockade of inhibitory/checkpoint receptors, or induction of enhanced functionality via cytokines (including ML differentiation) [7, 8, 23, 24]*.* In many cases, these therapeutic strategies benefit from large numbers of NK cells and multiple cycles of treatment, and hence several approaches have been developed to expand NK cells ex vivo for subsequent ACT. However, these methods typically require the use of non-GMP components and/or coculturing with irradiated tumor feeder cells. Such feeder cell-based approaches may be limited to early-phase clinical studies and are complicated by regulatory requirements related to characterization of these cells before and after irradiation and demonstrated removal of feeder cells and associated contaminants from the formulated NK cell product [23, 25–28]. To overcome these hurdles, we report here the development of a feeder cell-free two-step approach employing a previously described multifunctional fusion protein [17] to initiate ML programing in purified human NK cells ex vivo, followed by incubation with a second multifunctional fusion protein bound to an IgG1 antibody to facilitate signaling by CD16 and cytokine receptors on the NK cell surface, thereby expanding the ML NK cell population. We demonstrate that this novel feeder cell-free protein-based approach resulted in robust rapid multi-log expansion of ML NK cells for ACT against cancers.

## Materials and methods

### Mice

Female 6-to 8-week-old NSG mice (NOD-SCID gamma mice) (NOD.Cg-*Prkdc*^*scid*^* Il2rg*^*tm1Wjl*^/SzJ) were purchased from The Jackson Laboratory (Bar Harbor, ME). All animal husbandry procedures performed were approved by the Institutional Animal Care and Use Committee and were in accordance with the NIH Guide for the Care and Use of Laboratory Animals. Mice were maintained in a temperature/ humidity-controlled facility with 12-h light–dark cycles, group housed, and had free access to food and water. All mice were treated humanely throughout the experimental period.

### Construction and production of multifunctional protein complexes

HCW9206 is a complex of two fusion proteins, one comprising IL-7 linked together with the extracellular domain (aa 1–219) of human tissue factor (TF) and IL-15; and the other comprising IL-21 linked to the soluble domain of IL-15Rα (IL-15RαSu). The corresponding coding DNA sequences were synthesized (GENEWIZ, Inc., South Plainfield, NJ), cloned into pMSGV-1 modified expression vectors and transfected into CHO.K1 cells (ATCC, Manassas, VA). Co-expression of the two polypeptides in CHO cells allows for formation of the protein complex via high affinity interactions between the IL-15 and IL-15RαSu domains and secretion of the complexes into the culture media. HCW9206 expression was then detected with product-specific ELISA.

Production cell banks for HCW9206 were generated from stably transfected clonal cell lines following limited dilution cloning. Subsequent fusion protein complex production was conducted using fed-batch methods with chemically defined media in shake flasks or stir tank bioreactors. HCW9206 was purified from clarified culture media using immunoaffinity chromatography with anti-TF Ab-conjugated Sepharose resin, and then buffer-exchanged into PBS. A GMP-suitable manufacturing process (scaled from 2 to 200 L) was developed for HCW9206 consisting of immunoaffinity chromatography, low pH viral inactivation/depth filtration, multimodal chromatography, nanofiltration, and ultrafiltration/diafiltration steps employing commercially scalable methods. The purified HCW9206 product was characterized and released using qualified test methods per established specifications.

### Biological activity on cytokine dependent cell lines (IL-7, IL-15, and IL-21)

To assess the IL-7 IL-15, and IL-21 activities of the HCW9206 complexes, cytokine-dependent 2B8 (Sigma), CTLL-2 (ATCC), and B9 cells (Sigma), respectively, were used in combination with the presto blue proliferation assay per manufacturer’s instructions. Bioactivity of human recombinant cytokines IL-7, IL-15, and IL-21 were assessed as positive controls.

### NK cell isolation and culture

Healthy donor PBMC were collected from blood of anonymous, healthy, platelet donors (One Blood, Orlando, FL or local blood bank). NK cells (≥ 90% CD56^+^CD3^−^) were isolated using RosetteSep (STEMCELL Technologies) or Miltenyi MACS micro beads (Miltenyi Biotec), as described per manufacturer’s instructions. Isolated NK cells were cultured in RPMI-1640 (GIBCO), supplemented with 2 mM L-glutamine (Thermo Life Technologies), antibiotics (penicillin, 100 U/mL; streptomycin, 100 µg/mL) (Thermo Life Technologies), and 10% (v/v) fetal bovine serum (Hyclone) in the presence of either single cytokines, mixed-cytokines or HCW Biologics’ proprietary fusion molecules (HCW9201 [17] or HCW9206/HCW9101 complex). In some studies, NK cell controls were maintained in low-dose (LD) IL-15 (1 ng/mL).

### “Prime and Expand” method

Purified NK cells were counted and resuspended at 1 × 10^6^/mL to 1 × 10^8^/mL in complete media and then plated in flat bottom well plates or flasks. For the “Prime” method, cells were stimulated with either a mixture of cytokines: rhIL-12 (10 ng/mL) (Biolegend), rhIL-18 (50 ng/mL) (R&D Systems), and rhIL-15 (50 ng/mL) (R&D System) or HCW9201 complex (100 nM) for overnight at 37 °C. For the “Prime and Expand” method, “Prime” cells were diluted from 1 × 10^8^/mL to 2 × 10^6^/mL and treated with the complex formed by mixing HCW9206 (100 nM) and HCW9101 (anti-tissue factor antibody, αTF Ab) (50 nM) (HCW9206/HCW9101) in 24-well plates at 37 °C, 5% CO_2_. Cells were maintained at a density of 2 × 10^6^/mL with addition of fresh complete media containing HCW9206 (100 nM) and HCW9101 (50 nM). Cell expansion was measured based on fold expansion. For “Expand” only, purified NK cells were counted and resuspended at 2 × 10^6^/mL and treated with HCW9206 (100 nM) and HCW9101 (50 nM) (or in some experiments, anti-TF-IgG4) in a plate at 37 °C, 5% CO_2_. Cells were maintained at a density of 2 × 10^6^/mL to 0.25 × 10^6^/mL with fresh complete media containing HCW9206 (100 nM) and HCW9101 (50 nM) for up to 7–21 days, depending upon the scale of expansion.

For some studies, the treated NK cells were washed with HBSS without calcium, magnesium, or phenol red (Gibco) and resuspended in Human AB serum (Access Cell Culture LLC) with 10% DMSO for cryopreservation of 1 ml aliquots in cryovials. Cryovials were placed in freeze chambers to cool at a rate of 1 °C/min in a − 80 °C freezer and moved to storage in LN_2_ after 4–24 h.

### Metabolism assessment

To assess metabolism activity of “Prime” and “Prime and Expand” NK cells, cells were stimulated with either rhIL-15 (50 ng/mL) (R&D System) (control) or HCW9201 complex (100 nM) (“Prime”) for overnight at 37 °C. The “Prime” cells were diluted from 1 × 10^8^/mL to 2 × 10^6^/mL and treated with HCW9206 (100 nM) and HCW9101 (50 nM) (“Expand”) or rhIL-15 (50 ng/mL) (control) in 24-well plates at 37 °C, 5% CO_2_. Cells were maintained at a density of 2 × 10^6^/mL with fresh complete media containing HCW9206/HCW9101 or rhIL-15 up to 14 days.

Cells were harvested, counted, and resuspended in 4 × 10^6^/mL and then seeded at 50 µL/well in Cell-Tak-coated Seahorse Bioanalyzer XFe96 culture plates in Seahorse XF RPMI medium, pH 7.4 supplemented with 2 mM L-glutamine. The cells were allowed to attach to the plate for 30 min at 37 °C. An additional 130 µL of assay medium was added to each well of the plate (also the background wells). The plate was incubated at 37 °C for 1 h. For calibration plate, the 10 × solution of glucose/oligomycin/2DG was prepared in Seahorse assay media. A volume of 20 µL of either glucose (10 mM), oligomycin (2 µM), or 2DG (100 mM) were added to ports A, B, and C of the extracellular flux plate that was calibrated overnight. Extracellular acidification rate (ECAR) was measured using an XFe96 Extracellular Flux Analyzer. Complete ECAR analysis consisted of four stages: basal (without drugs), glycolysis induction (10 mM glucose), maximal glycolysis induction (2 μM oligomycin), and glycolysis inhibition (100 mM 2-DG).

#### FACS analysis of activation and intracellular (IFN-γ) markers

Purified NK cells maintained in rhIL-15, or treated with the “Prime,” “Expand” or “Prime and Expand” methods described above. For intracellular staining, cells were treated with 10 µg/mL of Brefeldin A (Sigma) and 1 × of Monensin (eBioscience) for 4 h. Cells were harvested and surface stained for CD56-BV421 (Biolegend), CD16-BV510 (Biolegend), CD25-APC (Biolegend), CD69-APCFire750 (Biolegend), CD62L-PeCy7 (Biolegend), and NKG2A (Biolegend) for 30 min. After surface staining, cells were washed (1500 RPM for 5 min in room temperature) in FACS buffer (1X PBS (Hyclone) with 0.5% BSA (EMD Millipore) and 0.001% sodium azide (Sigma)) and fixed for 10 min at room temperature. After fixation steps, cells were washed (1500 RPM for 5 min in room temperature) in 1 × Permeabilized Buffer (eBioscience) and stained for intracellular staining of IFN-γ-PE (Biolegend) for 30 min at room temperature. Cells were washed once again with 1 × Permeabilized Buffer and then washed with FACS buffer. Cell pellets were resuspended in 300 µL of FACS Buffer for analysis by flow cytometry (Celesta-BD Bioscience).

### CellTrace Violet labeling and analysis

Purified NK cells were resuspended in PBS 0.1% BSA at up to 2 × 10^7^ cells/mL and incubated with 5 μM CellTrace violet (CTV) (Invitrogen) at 37 °C for 20 min. CTV labeling was quenched with 5 mL ice cold R10 complete media. Cells were stimulated with different conditions as described in Fig. 3C. Cells were harvested after day 7 and were resuspended in 300 µL of FACS Buffer and cell proliferation was analyzed by flow cytometry (Celesta-BD Bioscience).

### Mouse model for assessing in vivo persistence of “Expand” and “Prime and Expand” NK cells

Purified NK cells treated with the “Expand” or “Prime and Expand” methods described above. The treated NK cells were washed three times in warm HBSS Buffer (Hyclone) at 1000 rpm for 10 min at room temperature. Cells were resuspended in HBSS buffer at a concentration of 10 × 10^6^ cells/0.2 mL and injected intravenously in tail-vein of NSG mice. In the animals, cells were supported with subcutaneous injections of Proleukin (IL-2) (5000 IU) or HCW9206 (3 mg/kg) every 48 h, for up to 12 days. Engraftment of cells was measured every week in blood staining for hCD45, mCD45, hCD56, hCD3, and hCD16 by flow cytometry (Celesta-BD Bioscience). For epigenetic and cell functional activity experiments, at Day 14, engrafted cells from the spleen were purified with the RosetteSep/human NK cell reagent (StemCell Technologies).

### Epigenetic analysis of DNA methylation

NK cells were isolated from fresh human leukocytes using the RosetteSep/human NK cell reagent (StemCell Technologies) as described above. The purified NK cells (2 × 10^6^ cells) were transferred to a 24-well flat bottom plate, and subjected to either: no treatment (control), or stimulated with mixture of cytokines: rhIL-12 (10 ng/mL) (BioLegend), rhIL-18 (50 ng/mL) (R&D Systems) and rhIL-15 (50 ng/mL) (R&D Systems) or HCW9201 (100 nM) for 15 h (overnight) at 37 °C, 5% CO_2_. NK cells were then washed and resuspended in complete RPMI 1640 medium supplemented with either low dose of rhIL-15 (1 ng/mL) or complex of HCW9206 (100 nM) and HCW9101 (50 nM) and incubated for 14 days. Cell cultures were replenished with fresh medium supplemented with either rhIL-15 (1 ng/mL) or a complex of HCW9206 (100 nM) and HCW9101 (50 nM), and cells were kept a density of approximately 10^6^ cells/mL.

DNA methylation of the distal IFNγ conserved noncoding sequence 1 (CNS-1) enhancer region containing six informative methylation-regulated CpG sites located at positions − 4399, − 4377, − 4360, − 4325, − 4293, and − 4278 relative to the transcription start site (Supplemental Fig. 1) was determined by pyrosequencing reactions as described elsewhere [17]. Briefly, genomic DNA was extracted from treated-NK cells using the QIAamp UCP DNA Micro Kit (Qiagen), and subjected (500 ng) to sodium bisulfite deamination using the EZ DNA Methylation-Direct kit (Zymo Research) according to the manufacturer’s protocol. This bisulfite treatment introduces methylation-dependent changes in the DNA with demethylated cytosines being converted into uracil, whereas methylated cytosines remain unchanged. Then, the bisulfite-treated nDNA (10—50 ng) was used as template to PCR amplify a 247 bp fragment of the *IFNG* CNS-1 region (dsPCR-CNS-1) containing the six informative CpG sites (Supplemental Fig. 1). The PCR was performed using the Pyromark PCR kit (Qiagen) with the forward primer IFNG-CNS1F (5’-AGAAAAGGGGGGATTTA-3’) and the biotinylated reverse primer IFNG-CNS1R-bio (5’-TAACACTCACAACCAAATTATC-3’) (GENEWIZ) (Supplemental Fig. 1). The PCR was performed at 95 °C for 15 min, 50 cycles of 35 s at 95 °C, at 55 °C for 35 s, at 72 °C for 40 s followed by at 72 °C for 5 min. The integrity and quality of the 247 bp PCR amplified products were visualized on a 1.2% TAE agarose gel. The pyrosequencing reactions were performed at Johns Hopkins University Genetic Resources Core Facility using the primers C4399-CNS1F (5’-GGGGATTTAGAAAAAT-3’, specific to the CpG sites −4399, −4377, −4360, and −4325), and C4293-CNS1F (5’-TGTATGATGTTAGGAGTTT-3’, specific to the CpG sites −4293, −4278, and −4227). Human non-methylated and methylated genomic DNA were used as DNA methylation controls (Zymo Research). The methylation percentages of the six informative IFNγ CNS-1 CpG sites were averaged for each treatment. Treatment of NK cells with HCW9201 followed by a low dose of rhIL-15 (1 ng/mL), shown to promote memory-like NK cell differentiation, was used as comparative controls [17]. Unexposed/untreated NK cells from donors were used as controls for DNA methylation levels prior to treatments.

### THP-1 tumor cell clearance in vivo

THP-1-Luciferase cells (Human Burkitt’s lymphoma) (ATCC) were cultured in complete media (RPMI 1640 (Gibco) supplemented with 2 mM L-glutamine (Thermo Life Technologies), penicillin (Thermo Life Technologies), streptomycin (Thermo Life Technologies), and 10% FBS (Hyclone). Tumor cells were washed three times in warm HBSS Buffer (Hyclone) at 1000 rpm for 10 min at room temperature. Cells were resuspended in 10 × 10^6^/0.2 mL HBSS buffer and injected intravenously in tail-vein of NSG mice. Engraftment of THP-1-Luciferase cells was assessed in NSG mice by bioluminescence imaging. “Expand” or “Prime and Expand” NK cells were washed three times in warm HBSS Buffer (Hyclone) at 1000 rpm for 10 min at room temperature. Cells were resuspended in 10 × 10^6^/0.2 mL HBSS buffer and injected intravenously in tail-vein of NSG mice. Transferred NK cells were supported with rhIL-2 (10 ng/ml) and rhIL-15 (10 ng/mL) by intraperitoneal (i.p.) injection every 48 h for up to 21 days. THP-1-Luciferase cells activity were assessed in NK cell-infused NSG mice by bioluminescence imaging. THP-1-Luciferase tumor-bearing mice without NK cell transfer served as controls. “Expand” and “Prime and Expand” infused NK cell numbers and activation markers were assessed cells isolated from blood at different time points by flow cytometry.

### Mass cytometry

Viably cryopreserved cells (> 70% recovery, > 90% viability) were thawed and immediately stained for mass cytometry, using validated and previously described methods [19, 21, 22] with the marker panel described in Supplemental Table 1. Stained samples were acquired on Helios mass cytometer (Fluidigm) and analyzed using Cytobank (Beckman Coulter). NK cells were gated using an unbiased approach from CD45^+^ events using FlowSOM: using all events, hierarchical consensus, 7 metaclusters, 49 clusters, 10 iterations, and a random seed on CD11b, CD16, CD19/CD3/CD14, CD45, CD56, and granzyme B. 3 NK clusters were defined as CD56^+^, Lineage-negative, CD45^+^, granzyme B^+^, CD11b^−/lo^, CD16^±^. Using this unbiased approach, NK cells represented 97.6% ± 4.2% (mean ± SD, *n* = 83) of all CD45^+^ events, which was expected. For visualizing the distinct populations, UMAP analyses were performed on NK cells, using all events on: CD11b, CD137, CD16, CD25, CD38, CD45, CD56, CD57, CD69, CD71, CD8, CD98, DNAM-1, EOMES, GLUT-1, granzyme-B, Ki-67, KIR2DL1_2DS1, KIR2DL2_2DL3, KIR2DL5, KIR2DS4, KIR3DL1, LAG-3, MIP1a, NKG2A, NKG2C, NKG2D, NKp30, NKp44, NKp46, NKp80, Perforin, T-bet, TGFBRII, TRAIL; “Num neighbors” = 15; minimum distance = 0.01, collapse outliers, and normalize scales.

### NK cell cytotoxicity assays

K562 luciferase cells (American Type Culture Collection, Manassas, VA) were cultured in RPMI 1640 medium supplemented with 10% FBS; 2 mM L-glutamine; 50 IU/mL penicillin; 50 μg/mL streptomycin. K562 luciferase cells were cultured with “Prime,” “Expand,” or “Prime and Expand” NK cells or rhIL-15-maintained cNK cells (controls) in different ratios for 24 h. Cell viability of K562 luciferase cells was assessed by flow cytometry (Celesta-BD Bioscience) [29].

### Granzyme B and IFN-γ ELISA

NK cells were assessed for their ability to secrete granzyme B and IFN-γ after tumor cell exposure. Briefly, NK cells were cultured for 24 h in IMDM supplemented with 10% FBS and rhIL-2 with K562-Luc cells at 2:1 E:T ratio. Supernatant was harvested and then analyzed using a Luminex assay (R&D systems) following the manufacturers protocol. Data are presented as pg/mL.

### Bioluminescence imaging

BLI was performed as described previously [17].

## Results

### Design and characterization of HCW9206, a fusion protein complex containing IL-7, IL-21, and IL-15 cytokines

**Fig. 1:**
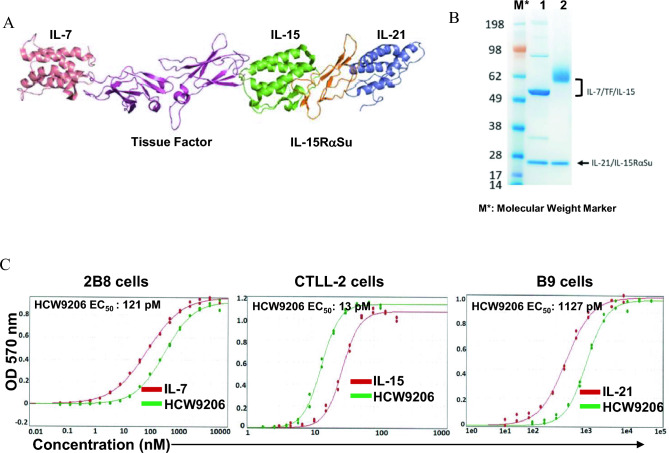
Biochemical characteristics of multifunctional fusion protein complex, HCW9206 **A** Ribbon diagram of HCW9206 comprising IL-21–TF-IL-15 and IL-7-IL-15Rα complex. **B** The recombinantly expressed HCW9206 polypeptides are highly glycosylated and therefore difficult to resolve by standard SDS-PAGE. To examine the protein characteristics, the proteins were deglycosylated and run on 4–12% SDS-PAGE Bis-Tis gels under reducing conditions and stained with InstantBlue. Lane1—deglycosylated HCW9206; Lane 2—non-deglycosylated HCW9206. **C** Dose response curves of HCW9206 (green) and control molecules (red) showing the concentration of HCW9206 required to activate individual cytokine dependent cell lines, 2B8 (IL-7 dependent), CTLL-2 (IL-15 dependent), and B9 (IL-21 dependent). Data are from two independent experiments (*n* = 2)

We previously reported the generation of novel multifunctional fusion protein complexes as immunostimulatory reagents and immunotherapeutics using a human tissue factor (TF)-based protein scaffold [17, 30]. In this study, we genetically fused human IL-7 to the N-terminus of the extracellular domain of human TF linked through its C-terminus to human IL-15. We also genetically fused the human IL-21 domain to the N-terminus of the extracellular human IL-15Rα sushi domain (IL-15RαSu). When co-expressed in recombinant CHO cells, these fusion proteins formed a soluble heterodimeric complex, designated as HCW9206, through high affinity interactions between the IL-15 and IL-15RαSu domains (Fig. 1A). The CHO expressed fusion protein complex is secreted into the cultured media and can be readily purified using anti-TF antibody (Ab)-based affinity chromatography, as described previously [17, 30]. Based on reducing SDS-PAGE analysis, the molecular weight of the deglycosylated IL-7/TF/IL-15 and IL-21/IL-15RαSu polypeptides were ~ 50 kDa and ~ 25 kDa, respectively, as expected based on the amino acid sequences (Fig. 1B).

Using the purified protein complex, we evaluated whether HCW9206 retained the biological activities of its component cytokines. Since human IL-15, IL-21, and IL-7 promote the growth of murine CTLL-2 [31], B9 [32], and 2E8 cells [33], respectively, we stimulated each of these cell lines with individual cytokines (controls) or HCW9206. We observed that the IL-15, IL-21, and IL-7 components of HCW9206 were able to promote proliferation of the responsive cells with EC_50_’s of approximately 13 pM, 1127 pM, and 121 pM, respectively (Fig. 1C). Interestingly, HCW9206 exhibited twofold greater IL-15 activity than recombinant IL-15, potentially due to the formation of the IL-15/IL-15RαSu complex with enhanced biological activity. The IL-7 and IL-21 components of HCW9206 appeared to have ~ threefold lower activity than the free cytokines, potentially due to fusion of the C-termini of these cytokines to other polypeptides in HCW9206. Together, these findings indicate that the biological activities of IL-7, IL-15, and IL-21 were retained in the HCW9206 heterodimeric complex.

### HCW9206 in combination with an anti-tissue factor IgG1 antibody led to expansion and activation of NK cells from human peripheral blood

**Fig. 2:**
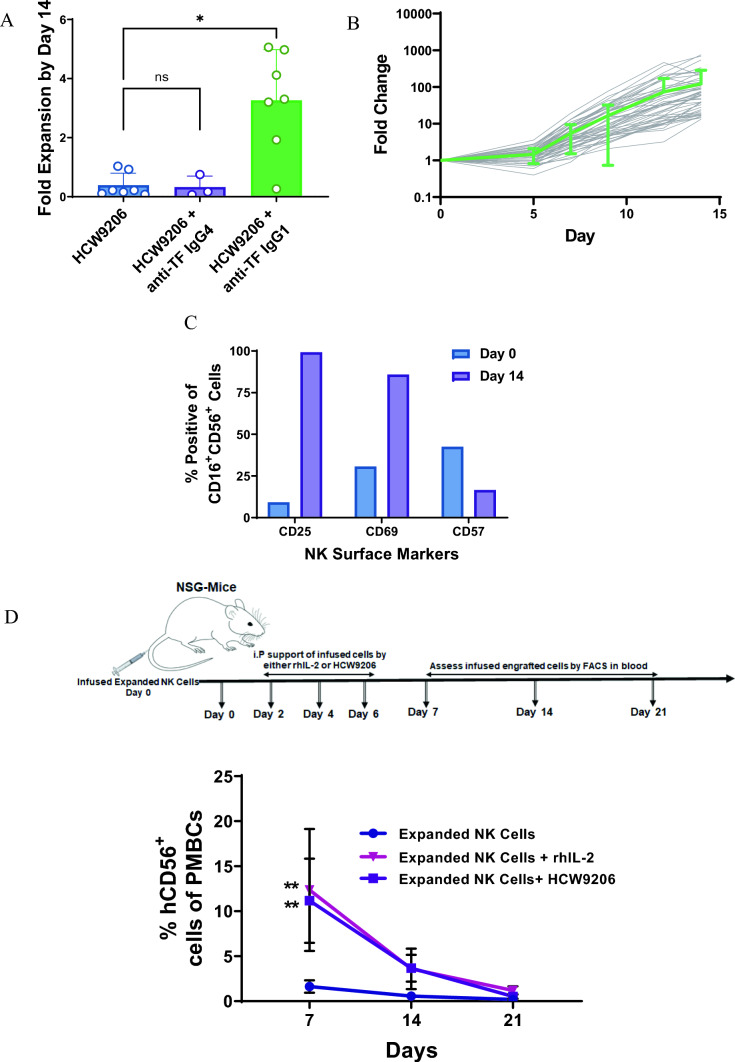
Expansion of CD56^+^ NK cells ex vivo. **A** Short term expansion of human NK cells in media containing HCW9206 with or without anti-TF IgG1 or IgG4 antibodies. Data shown (mean ± SD) is from 7 donors analyzed in 2 independent experiments. (ANOVA, post-hoc Tukey, *< 0.05). **B** Expansion of NK cells purified from PBMC of 15 donors with 3 independent replicates each. NK cells were expanded in the presence of HCW9206/HCW9101 complex from day 0 to day 14 of culture. Individual expansion data are indicated by grey lines. Green line is the mean ± SD of all expansions shown. **C** Change in CD25, CD69 and CD57 markers on NK cells before and after Day 14 expansion in media containing HCW9206/HCW9101 complex. **D** Persistence of HCW9206/HCW9101-expanded NK cells in NSG mice. Top–study scheme, 10 × 10^6^ HCW9206/HCW9101-expanded cells were infused in tail vein of NSG mice. Infused cells were supported by either rhIL-2 or HCW9206 by every other day for 6 days. Bottom—blood was assessed for presence of infused NK cells on days 7, 14, and 21. Data represent means ± SD (*n *= 3) (2-way ANOVA, **< 0.01 vs. expanded NK cell control)

Studies have shown that various combinations of IL-2, IL-15, IL-18, and IL-21 were able to expand human NK cells ex vivo [28, 34, 35]. Additionally, IL-7 has been reported to promote survival of human CD56^bright^ NK cells [36, 37]. Thus, we freshly purified CD3^−^ CD56^+^ NK cells from human donor peripheral blood (PB) and compared their growth in media containing HCW9206. However, in initial studies, we found that HCW9206 alone did not support significant expansion of purified human NK cells (Fig. 2A). Since CD16 Fc receptor engagement with immobilized IgG1 antibody (Ab) or anti-CD16 Ab was previously shown to provide a second signal that promotes cytokine-driven human NK cell expansion ex vivo [38, 39], an IgG1 Ab (HCW9101) recognizing the soluble TF scaffold domain of HCW9206 was used to pre-form a complex with HCW9206 (i.e., HCW9206:HCW9101 = 2:1 in the mixture). When compared to incubation with HCW9206 alone, addition of the HCW9206/HCW9101 complex supported a significant increase in the expansion of purified human NK cells (Fig. 2A). To demonstrate that engagement of IgG1 with CD16 on NK cells was essential for providing a proliferative signal, we converted HCW9101 from an IgG1 to an IgG4 subclass. The anti-TF-IgG4 antibody would not be expected to bind to the Fc receptor displayed on the human NK cells and, when compared to HCW9101 IgG1, the complex of HCW9101 IgG4 and HCW9206 did not support cell expansion in any of the same donor NK cell preparations (Fig. 2A).

We further optimize the conditions for ex vivo NK cell growth by maintaining cell concentrations at 2 × 10^6^/mL through dilution every other day with fresh media containing 100 nM HCW9206 and 50 nM HCW9101. We then used this method to expand NK cells purified from the peripheral blood of 15 individual donors, across three independent experiments. Cell counts increased about 1.4-fold over the first 5 days in culture, and then cells entered a rapid growth phase and increased exponentially over the next 10 days. By day 14, the NK cells expanded an average of 120-fold with a range of 15- to 800-fold depending on the donor (Fig. 2B). On average, the NK cells doubled about every 32 h during the expansion phase, with NK cells from certain donors doubling every 24 h. For individual donors, the responsiveness of different NK cell preparations to HCW9206/HCW9101 was reproducible but the underlying properties responsible for variations in response among different donors remain under investigation. In addition to cell proliferation, ex vivo incubation with HCW9206/HCW9101 complex for 14 days induced NK cell expression of activation markers CD25 and CD69 whereas CD57 expression decreased (Fig. 2C). Together, these findings show that the combined signaling of IL-7, IL-15, and IL-21 receptors and CD16 engagement by the HCW9206/HCW9101 complex was sufficient to stimulate robust human NK proliferation ex vivo without the need for exogenous feeder cells.

### HCW9206/HCW9101 complex activates human NK cells with prolonged in vivo persistence with cytokine support

We then evaluated the ability to adoptively transfer and support HCW9206/HCW9101-expanded human NK cells in mice. NSG (NOD/SCID gamma) mice were intravenously infused with 2 × 10^6^ human NK cells that had been grown for 14 days in media containing HCW9206/HCW9101 complex. The mice were subsequently treated subcutaneously with recombinant human IL-2 (rhIL-2) or HCW9206 (or no treatment) every 2 days for a total of 3 doses as cytokine support for immune cell maintenance. We found that compared to NSG mice with no cytokine support, treatment with either rhIL-2 or HCW9206 resulted in elevated peripheral blood levels of the adoptively transferred ex vivo expanded human NK cells (Fig. 2D).

### “Prime and Expand” strategy to activate and expand human ML NK cells

**Fig. 3:**
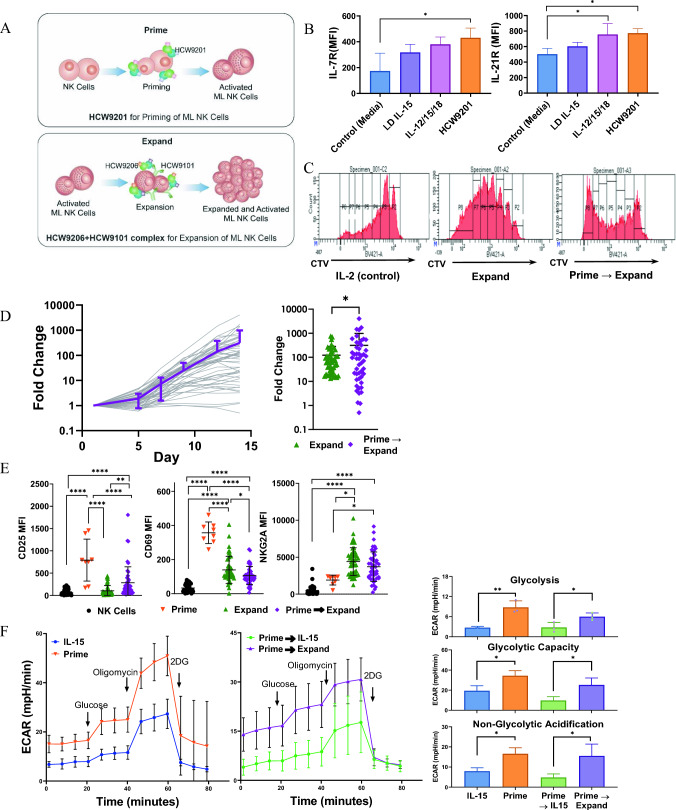
Characterization of NK cells incubated ex vivo with multifunctional fusion protein complexes. **A** Model of “Prime” of NK cells utilizing HCW9201 and “Expansion” of “Prime” NK cells with HCW9206/HCW9101 complex. **B** Bar graph showing expression of IL-7R and IL-21R on NK cells after overnight stimulation by either low-dose (LD) IL-15, mixed cytokines of IL-15, IL-12, and IL-18 or HCW9201 (*n* = 3 donors). **C** Purified NK cells were labeled with CellTrace Violet dye and incubated for 7 days with rhIL-2 (10ng/ml) or HCW9206/HCW9101 (“Expand”) or HCW9201 followed by HCW9206/HCW9101 (“Prime and Expand”) Cells were then harvested and examined for CellTrace Violet dye dilution by flow cytometry for cell proliferation. Percentage of cells divided was determined by FlowJo Proliferation Modeling (v. 10.3). Data are representative of 2 independent experiments. **D** Left—Expansion of NK cells purified from PBMC of 15 donors with 3 independent replicates each. NK cells were primed overnight in the presence of HCW9201 on day 0 followed by HCW9206/HCW9101 expansion from day 1 to day 14 of culture. Individual expansion data are indicated by grey lines. Purple line is the mean ± SD of all expansions shown. Right—Total fold expansion of “Expand” and “Prime and Expand” treated NK cells after 14 days of culture (Paired *t*-test, *< 0.05). **E** Surface protein expression of CD25, CD69, and NKG2A measured by flow cytometry of isolated NK, “Expand” NK cells, “Prime and Expand” NK cells. Cells were thawed from cryopreservation and then stained with antibodies to CD45, CD56, CD3, Viability, CD25, CD69, and NKG2A. NK cells were gated as non-Debris, single cell, CD45^+^, CD56^+^CD3^−^, Live cells and shown as mean ± SD of the MFI. (ANOVA, post-hoc Tukey, *< 0.05, **< 0.01, ****< 0.0001). **F** Metabolic parameters were examined for resting, IL-15 (control), “Prime” alone, “Prime” and supported by rhIL-15 and “Prime and Expand” NK cells using XFe96 Extracellular Flux Analyzer measurements of extracellular acidification rate (ECAR). Left—complete ECAR analysis consisted of four stages: basal (without drugs), glycolysis induction (10 mM glucose), maximal glycolysis induction (2 μM oligomycin), and glycolysis inhibition (100 mM 2-DG). Right—summary data (mean ± SD) showing glycolysis, glycolytic reserve, and glycolytic capacity. Data shown is from 6 donors analyzed in 3 independent experiments (n = 5). (ANOVA, *< 0.05, **< 0.01)

Clinical studies are currently underway to evaluate adoptive transfer of rapid point-of-care manufactured ML NK cells with promising results reported for hematological malignancies [19, 21, 22]. However, one limitation of employing ML NK cells in this fashion is their fixed numbers (0.5 − 1.5 × 10^9^) per apheresis, resulting in the inability to test higher cellular doses and repeat dosing schedules. Since the HCW9206/HCW9101 complex expands human NK cells, we hypothesized that an ex vivo “Prime and Expand” strategy could generate a large number of ML NK cells without the use of feeder cells. In this process, purified human NK cells are first incubated overnight with HCW9201 to initiate the ML NK cell program via IL-12/15/18 receptors (“Prime” step), followed by incubation with HCW9206 and HCW9101 (“Expand” step) to expand the ML NK cells over a 2-week period (Fig. 3A).

Overnight stimulation of human NK cells with IL-15, a cocktail of IL-12, IL-15 and IL-18, or HCW9201 resulted in increased expression of receptors for IL-7 and IL-21, supporting the idea of expansion using the HCW9206 combination of cytokines (Fig. 3B). We further evaluated this with purified human NK cells from peripheral blood (PB) that were first labeled with CellTrace Violet and then grown in IL-2 (Control), expanded in HCW9206 and HCW9101 (“Expand”), or stimulated overnight with HCW9201 and then expanded in HCW9206 and HCW9101 (“Prime and Expand”). After 7 days, proliferation of the NK cells was measured by dye dilution. As shown in Fig. 3C, most of the PB NK cells underwent one cell division when maintained in IL-2 and 3 to 4 divisions when expanded in HCW9206 and HCW9101. NK cells that were first stimulated with HCW9201 and then expanded in HCW9206/HCW9101 complex proliferated the most, showing up to 6 cell divisions (Fig. 3C). When “Prime and Expand” methods were used on NK cells from 15 individual donors, the average cell expansion was over 300-fold over 15 days, with a range of 0.5-fold to greater than 4,000-fold expansion depending on the donor cells (Fig. 3D). When compared to “Expand” only, “Prime and Expand” cultures showed greater variability in cell proliferation rates, but the overall fold expansion was significantly higher (Fig. 3D), confirming that HCW9201 induced greater responsiveness to subsequent HCW9206/HCW9101-driven proliferation. Consistent with the results described above and previously [17], “Prime,” “Expand,” and “Prime and Expand” treatments all significantly induced ML markers (CD25, CD69, NKG2A) on NK cells when compared to control (Fig. 3E), though variations between these changes were seen with the different methods and donors.

Metabolism plays a key role in NK cell responses with cytokine stimulation impacting both glycolysis and oxidative phosphorylation [40]. To assess treatment mediated effects on metabolism, extracellular flux assays were performed on human NK cells following overnight activation with IL-15, or HCW9201, or following overnight activation with HCW9201 and then followed by 14-day expansion with IL-15 or HCW9206/HCW9101 complex. As previously shown [17], HCW9201 activation significantly increased rates of glycolysis, glycolytic capacity, and non-glycolytic acidification of human NK cells compared to IL-15 treatment. Interestingly, we also observed that “Prime and Expand” human NK cells exhibited enhanced glycolytic parameters (Fig. 3F) compared to HCW9201-activated NK cells maintained in low-dose IL-15.

### “Prime and Expand” human NK cells exhibit potent cytotoxic activity against tumor cells

**Fig. 4:**
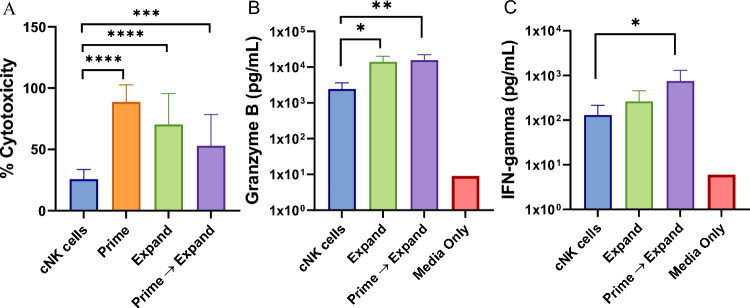
Cytotoxicity activities of “Prime and Expand” NK cells against K562 tumor targets. **A** Cytotoxic capacity of “Prime and Expand” NK cells were assessed from 7 donors with 3 independent replicates each, against K562 luciferase tumor cells (3:1 E:T ratio) in a 24-h assay. After 24 h, cells were lysed and luminescence of remaining K562 cells was measured. Mean ± SD are shown (ANOVA, ****< 0. 0001). **B** Granzyme B and IFN-γ secretion by NK cells measured by Luminex analysis of supernatants harvested from culture with K562 target cells at a 2:1 E:T ratio for 24 h. Mean ± SD (n = 5) is shown (ANOVA, *< 0.05, **< 0.01)

The cytotoxic activity of human NK cells generated with the “Prime and Expand” method was also evaluated against K562 leukemia targets. K562 cells expressing luciferase were incubated with cNK cells (Unstimulated NK cell) or “Prime,” “Expand,” and “Prime and Expand” NK cells, and cytotoxicity was assessed over a range of effector: target (3:1, E:T) ratios measured by loss of target signal. In each case, NK cells generated by the “Prime,” “Expand,” or “Prime and Expand” method exhibited significantly increased cytotoxicity compared to purified IL-15-supported NK cell controls (Fig. 4A).

The “Prime and Expand” NK cells also expressed higher levels of granzyme B (Fig. 4B) and IFNγ (Fig. 4C) following in vitro incubation with K562 cells when compared to K562-stimulated IL-15-supported NK cells. Thus, the “Prime and Expand” strategy was highly effective at rapidly expanding purified human NK cells with a ML phenotype and enhanced metabolic, cytotoxic, and activation responses.

### Phenotype evaluation of “Prime”, “Expand” and “Prime and Expand” ML NK cells

**Fig. 5:**
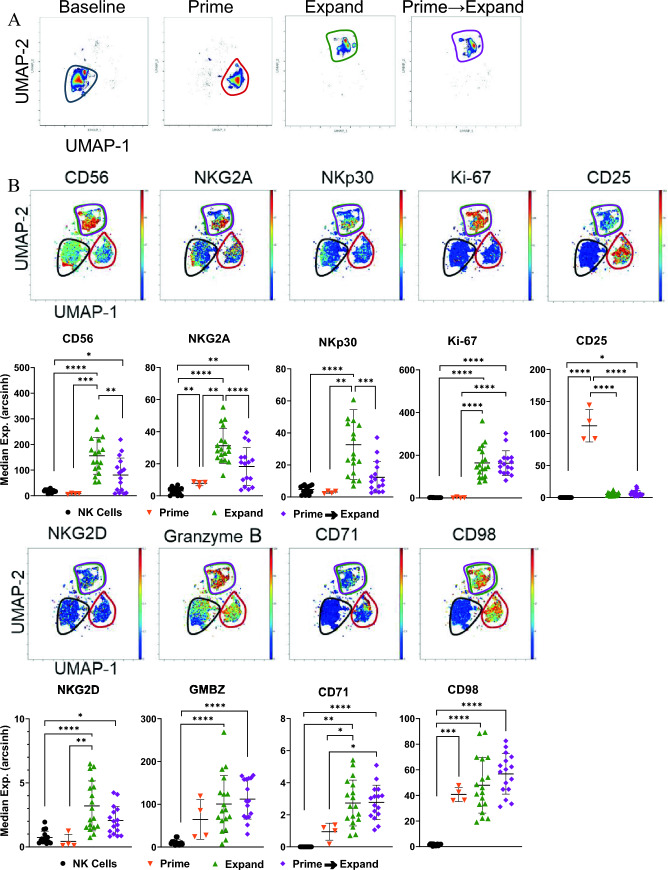
CyTOF analysis of “Prime,” “Expand,” and “Prime and Expand” NK cell phenotypes. **A** NK cells were assessed for their phenotype by CyTOF. Cryopreserved NK cells were run on a Helios mass cytometer and assessed using FlowSOM clustering on CD11b, CD16, CD19^-^ CD3^-^ CD14^-^ (LIN); CD45, CD56, GzmB. NK cells were identified across 3 metaclusters as LIN^-^ CD56^+^, CD16^±^, CD11b dim/- corresponding with isolated NK cells, “Prime” NK cells and “Prime and Expand” NK cells. Representative U-MAPs are shown for baseline (*n* = 15), “Prime” (*n* = 4), and “Prime and Expand” NK cells (*n* = 15).** B**. Overlay colored UMAPs by the indicated markers with median expression summarized below. Comparisons by ordinary one-way or Kruskal–Wallis as appropriate, determined by Shapiro–Wilk Normality test. Median expression was assessed on a Helios mass cytometer for each marker indicated from each metacluster (ANOVA, *< 0.05, **< 0.01, ***< 0.001, ****< 0.0001)

Multi-dimensional CyTOF analysis was also performed to further assess effector, memory, and maturation markers of human NK cells that had been treated with HCW9201 alone, HCW9206/HCW9101 complex alone, or HCW9201 followed by HCW9206/HCW9101 complex. Representative UMAPs of control, “Prime” and “Prime and Expand” human NK cells revealed distinct clusters based on the ex vivo treatment (Fig. 5A). Consistent with the flow cytometry data (Fig. 3E), NKG2A levels were elevated and CD25 levels were lower in “Expand” and “Prime and Expand” compared to “Prime” NK cells (Fig. 5B). Additionally, CyTOF analysis showed an increase in CD56^bright^ cells in NK cells from some donors as well as elevated NKp30 and Ki67 following “Expand” and “Prime and Expand” treatment. The increase in Ki67 was consistent with our observations that the human NK cells were viable and proliferative. We also observed that “Prime and Expand” NK cells upregulated maturation markers, NKG2D and granzyme B, and nutrient transporters, CD71 and CD98 (Fig. 5B). Collectively, these data demonstrate that “Prime and Expand” NK cells exhibited enhanced ML, maturation, and metabolic markers.

### “Prime and Expand” methods induced epigenetic demethylation of the *IFNG* CNS-1 region in human NK cells

**Fig. 6:**
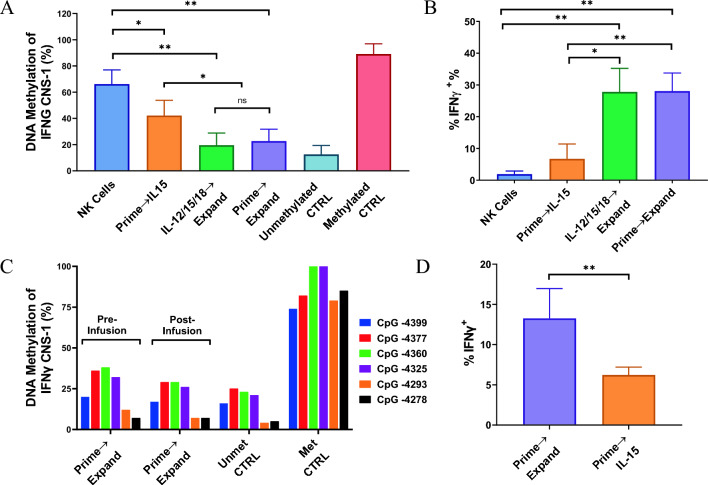
“Prime and Expand” method induces epigenetic remodeling of DNA methylation within IFNγ CNS-1 region of NK cells. **A** The DNA methylation status of six informative CpG sites within the CNS-1 region of the IFNγ gene promoter (located at − 4399,  − 4377, − 4360, − 4325,  − 4293, and − 4278 relative to the IFNγ transcription start site) was analyzed by pyrosequencing of bisulfite-treated DNA isolated from purified and treated NK cells obtained from human subjects (*n* = 4) as described in Materials and Methods. The NK cells were either untreated (NK cells) or stimulated with a mixture of single cytokines (hIL-12, hIL-15, hIL-18) or HCW9201 (“Prime”) for 12–16 h. Then, expanded for 14 days in presence of either a low dose of hIL-15 (IL-15, 1.0 ng/mL) or HCW9206/HCW9101 (“Expand”) with frequent replenishment of medium containing fresh agents to keep the cell density at approximately 10^6^ cells/mL. For controls, human unmethylated and methylated genomic DNA were used as templates in bisulfite and pyrosequencing reactions. The mean percentage of DNA methylation is depicted for each treatment. ANOVA of DNA methylation data (ns, not significant; *< 0.05; **< 0.01) using the GraphPad Software Prism 8 (Version 8.3.0). **B** IFNγ expression (% positive) in these activated NK cells as determined by flow cytometry analysis. **C** The DNA methylation changes associated with activated NK persists in vivo. Purified NK cells isolated from human subject (*n* = 1) were stimulated using the “Prime and Expand” strategy injected into NSG mice (*n* = 5) followed by s.c. HCW9206 for cytokine support. After nine days in the animals, the spleen of each mouse was collected, and NK cells were purified. The pre-infusion and post-infusion NK samples were subjected to pyrosequencing analysis of DNA methylation. The mean % DNA methylation for each of the six IFNγ CNS-1 CpG is shown. Low levels of DNA methylation were observed in the pre- and the post-infused NK cells. Human unmethylated (Unmet) and methylated (Met) genomic DNA were used as controls. **D** IFNγ (%) expression in harvested infused “Prime and Expand” and “Prime and IL-15 treated” NK cells, which were stimulated for 4 h with rh-IL-12/IL-15/IL-18. IFNγ expression (% positive) in these cells was measured by flow cytometry analysis. Data represent mean ± SD (*n* = 5) (*t*-test, **< 0.01)

We have previously shown that human NK cell stimulation with HCW9201 resulted in DNA methylation remodeling of the IFNγ conserved noncoding sequence 1 (CNS-1) region observed after ML differentiation [17]. To determine the epigenetic effects of the “Prime and Expand” method on DNA methylation of the CNS-1 region in NK cells, genomic DNA was extracted from unstimulated human NK cells or NK cells stimulated overnight with either HCW9201 or mixed cytokines of IL-12, IL-15, and IL-18 followed by growth for 14 days in the presence of HCW9206/HCW9101 or IL-15. The DNA was then subjected to pyrosequencing analysis and levels of DNA methylation were determined for each of the six CpG sites within the CNS-1 region (Supplemental Fig. 1). As shown in Fig. 6A, low levels of DNA methylation (% ^5m^CpG, averaged for 6 sites) were observed in the CNS-1 region obtained from NK cells generated with the “Prime and Expand” method or following stimulation with the cytokine cocktail and expansion in HCW9206/HCW9101, as compared to unstimulated NK cells or “Prime” NK cells maintained in a low dose of IL-15. The level of DNA demethylation observed at CpG sites within the *IFNG* CNS-1 region correlated with the level of IFNγ protein expression in these activated NK cells as evidenced by flow cytometry analysis (Fig. 6B). Taken together, these results suggest that long-term ex vivo exposure of NK cells to HCW9206/HCW9101 induced significant DNA demethylation of the CpG sites within the CNS-1 region, leading to epigenetic remodeling of the *IFNG* locus.

To evaluate whether these epigenetic changes persisted in vivo, NSG mice were injected intravenously with “Prime and Expand” ML NK cells followed by IL-2 support for 9 days. The engrafted NK cells were isolated and subjected to flow cytometry and epigenetic analyses. The level of DNA methylation at each of the six informative CpG sites within the *IFNG* CNS-1 distal promoter region was analyzed. The results revealed that the levels of DNA methylation at the CpG sites from post-infused NK cells were low and comparable to the levels of DNA methylation observed in the pre-infused NK cells (Fig. 6C). Additionally, these methylation levels were also similar to those observed in the unmethylated DNA control (Fig. 6C), confirming persistence of low levels of DNA methylation at these CpG sites. As expected, high levels of DNA methylation were observed with the methylated DNA controls. Persistence of CNS-1 region demethylation also correlated with the ability of the post-infusion “Prime and Expand” NK cells to express IFNγ compared to the “Prime—IL-15 treated” NK cells upon restimulation with IL-12/IL-15/IL-18 (Fig. 6D), consistent with the results of the pre-infusion NK cell shown in Fig. 6B. Taken together, these results indicate that the ML DNA methylation patterns of “Prime and Expand” NK cells are maintained and persist in vivo.

### “Prime and Expand” human NK cells show efficacy against THP-1 leukemia tumors and persist long-term in vivo

**Fig. 7:**
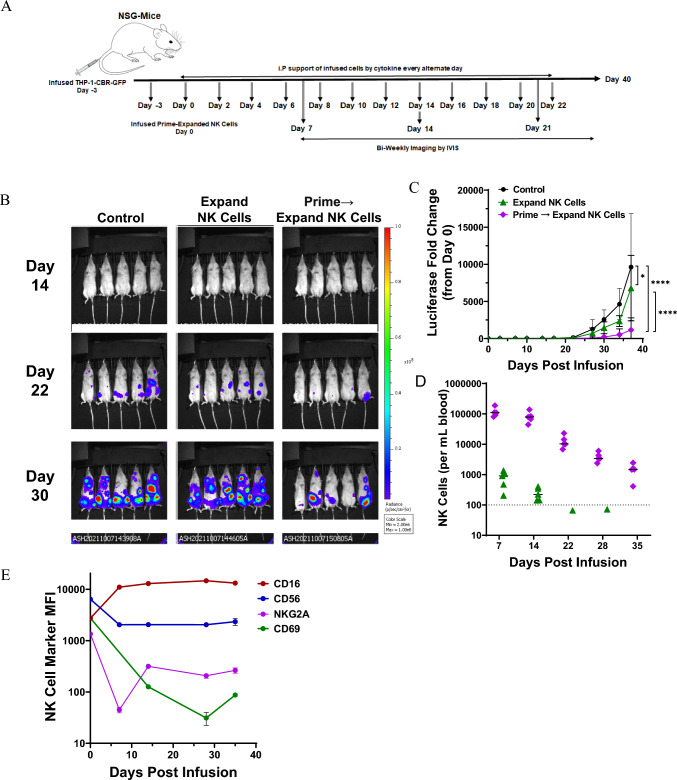
In vivo antitumor activities of “Expand” and “Prime and Expand” NK cells against THP-1 cells in NSG mouse model. **A** Schematic of the in vivo model used. A single dose of **“**Expand” or **“**Prime and Expand” (1 × 10^7^) NK cells was injected into NSG mice bearing THP-1-CBR-GFP tumor cells. Mice bearing THP-1 tumors without NK cell transfer served as controls. rhIL-2 and rhIL-15 support was infused every other day. **B** Efficacy of injected NK cells was assessed by bioluminescent imaging (BLI) of mice bearing THP-1 tumors. **C** Changes in tumor BLI were plotted over time. Significant difference in tumor size was observed between different treatments at D37 demonstrating **“**Expand” or “Prime and Expand” NK cells effectively reduced tumor burden relative to vehicle control (Two-way ANOVA, post-hoc Tukey, *< 0.05, ****< 0.0001). **D** Long-term persistence of NK cells in the mouse blood was assessed by flow cytometry. Gating Scheme: Singlet\Viable\GFP-ve\huCD45 + ve\mCD45-ve\CD56 + ve\CD3-ve. “Prime and Expand” NK cells demonstrated long-term NK cell persistence in the peripheral blood. **E** Phenotypic characterization of persistent NK cells was assessed by flow cytometry. Levels of surface markers are expressed as MFI (mean ± SD) over time. Gating Scheme: Singlet\Viable\GFP-ve\huCD45 + ve\mCD45-ve\CD56 + ve\CD3-ve

We employed an NSG (NOD-SCID IL-2Rγnull) mouse model with THP-1-GFP-Luc human leukemia monocytic cells [41] to evaluate the antitumor activity of human NK cells generated with both the “Expand” and “Prime and Expand” methods. THP-1 tumor cells were injected intraperitonially into the NSG mice and 1 × 10^7^ “Expand” or “Prime and Expand” NK cells were infused i.v. 3 days later. Tumor-bearing mice without transferred NK cells served as controls. In addition, alternate-day low dose of IL-2 or IL-15 was administered subcutaneously as shown in the schematic figure (Fig. 7A). Mice were imaged bi-weekly until day 35. Treatment with both “Expand” and “Prime and Expand” NK cells significantly decreased THP-1 tumor bioluminescence when compared to tumor-bearing mice without NK cell therapy (Fig. 7B and C, Supplemental Figs. 2 and 3). Moreover, we found that the “Prime and Expand” NK cells exhibited significantly greater antitumor activity than NK cells treated only with the “Expand” method (Fig. 7C), with decreased tumor burden seen as early as day 7 after NK cell transfer (Supplemental Fig. 3).

We also analyzed the persistence and activation markers of the infused NK cells isolated from blood collected every 7 days from the tumor-bearing mice. Infused human NK cells were tracked based on CD45^+^CD56^+^CD3^−^ staining by flow cytometry. We observed that “Prime and Expand” NK cells persisted in the circulation for at least 35 days post-infusion whereas “Expand” NK cells reduced to baseline levels by 22 days after adoptive transfer (Fig. 7D). We also observed that “Prime and Expand” NK cells-maintained activation markers CD16, CD56, NKG2A and CD69 (Fig. 7E). Collectively, these data show that “Prime and Expand” NK cells are active with improved persistence after adoptive transfer and are efficacious against tumors in the THP-1-GFP-Luc human leukemia mouse model.

## Discussion

Based on the unique properties and promising clinical activity of adoptively transferred NK cells, a variety of different approaches have been employed to generate allogeneic NK cell-based therapeutics. These include the use of different tissue sources from which NK cells can be derived, such as donor peripheral blood, cord blood, hematopoietic stem, and progenitor cells, and induced pluripotent stem cells. Details on the generation of clinical-grade NK cell products from these tissues, their advantages and disadvantages, and use of these products (and CAR engineered derivatives) in clinical studies have been recently reviewed [7, 8, 23, 28]. Peripheral blood (PB) derived-NK cells are highly functional but show intra-donor variability and are limited in the number that can be obtained by donor apheresis. Thus, donor selection and expansion of suitable NK cells is required to achieve an “off-the-shelf” product for multidose treatment of patients.

Various cytokine combinations have been used to expand PB NK cells while preserving their cytotoxic activity. However, cytokine-induced expansion has been generally limited to less than 20-fold over a 2-week period in culture [28]. Approaches using irradiated allogeneic or modified feeder cells (or membrane fragments from such cells) also provide essential stimulatory signals for NK cell proliferation [26]. For example, the leukemic K562 cell line has been engineered to express 4-1BBL and membrane bound IL-15 or IL-21 (K562-mb15-41BBL and K562-mb21-41BBL, respectively). Donor NK cells co-incubated with irradiated K562-mb15-41BBL or K562-mb21-41BBL were shown to proliferate 200- to 2000-fold in 14 days without loss of their activated phenotype or cytotoxicity [42]. While ex vivo treatment of donor NK cells with either cytokines or modified feeder cells result in cellular activation, the phenotype and functionality of the activated NK cells differ with respect to levels of inhibitory and activating receptors (i.e., KIR, CD16, NKp30, NKp46) [18, 42]. Such feeder cell-based approaches are also complicated by regulatory requirements to establish the sterility, identity, viability and potency of the feeder cells before and after irradiation in order to maintain consistency and eliminate infectious agents and viable tumor cells from the manufacturing process. Additionally, removal of residual feeder cells and their DNA and membrane fragments, a tedious manufacturing step, needs to be addressed during release testing of the formulated NK cell product [25–27].

In the study described here, we developed a new approach to activate and expand human PB NK cells using a multi-functional cytokine fusion protein/antibody complex. This approach provides the advantages of using well-characterized soluble GMP protein reagents to achieve up to 800-fold expansion of donor PB NK cells over a 2-week ex vivo culture period. We found that a fusion protein complex comprising IL-15, IL-21, and IL-7 domains (HCW9206) together with a human IgG1 antibody (HCW9101) specific to the scaffold domain of the fusion protein was most effective in promoting NK cell expansion ex vivo. Previous studies using human NK cells reported that IL-15 in combination with IL-21 can promote ~ fourfold expansion after 2–3 weeks in culture and that immobilized IgG1 antibody can augment cytokine expansion by ~ 2.5 fold [39, 43]. Thus, the level of NK cell expansion with HCW9206/HCW9101 represents a significant improvement on previous protein-based strategies. The HCW9206/HCW9101 method permits removal of the protein reagents in post-expansion wash steps and is readily amenable to scale-up. We postulate that in addition to pro-proliferative signaling through CD16 and the IL-15 and IL-21 receptors, HCW9206/HCW9101 promotes survival of the activated human NK cells via IL-7 receptor engagement [36, 37].

We also combined methods to activate memory-like responses in NK cells with subsequent HCW9206/HCW9101 expansion. In previous studies, ML NK cells were induced by overnight incubation of PB NK cells with IL-12, IL-15 and IL-18 or a multifunctional cytokine fusion protein, HCW9201 [9, 17]. These cells were found to exhibit enhanced effector functions in response to restimulation, as well as potent antitumor activity and prolonged persistence in vivo. ML NK cells have also shown clinical efficacy against relapsed/refractory hematological cancers in the pre- and post-transplant settings [18, 44]. We found that overnight stimulation of human NK cells with HCW9201 resulted in increased expression of IL-21 and IL-7 receptors and enhanced proliferation when subsequently incubated with HCW9206/HCW9101. This “Prime and Expand” method resulted in an average of 300-fold and up to 4,000-fold expansion of NK cells over a 2-week incubation period.

Elevated IFNγ production is one hallmark of the ML NK cells. It has been shown that *IFNG* expression is controlled by two CNS regions. *IFNG* CNS-2 is demethylated in peripheral blood mature NK cells suggesting the NK cells, unlike the naive T cells, maintain a basal level of *IFNG* expression. *IFNG* CNS-1 is heavily regulated by DNA methylation and is critical in NK cell differentiation since it responds to cytokine stimulation via demethylation [17, 45, 46]. Here, we demonstrated that the six informative CpG sites within the CNS-1 region of NK cells were significantly demethylated following the “Prime and Expand” treatment. These epigenetic imprints were stably maintained after adoptive transfer into NSG mice highlighting the persistency of these activated NK cells for ACT. Another aspect of ML NK cells that is maintained and improved in “Prime and Expand” NK cells is energy metabolism, where these cells exhibited significant increases in steady state glycolysis, glycolytic capacity, and non-glycolytic acidification. Finally, when adoptively transferred into NSG mice bearing THP-1-GFP-Luc human leukemia tumors, “Prime and Expand” NK cells significantly reduced tumor growth. Overall, “Prime and Expand” NK cells provided potent antitumor activity and long-term persistence while maintaining an activated phenotype.

Our findings are consistent with a previous report where purified human cord-blood NK cells were first activated overnight with IL-12/IL-15/IL-18 to induce ML properties and then expanded for 14-days in media containing IL-2 and irradiated K562 feeder cells engineered to express CD48, 4-1BBL, and membrane-bound IL-21 for optimal NK cell growth [47]. Pre-activation of cord-blood NK cells with the cytokine cocktail followed by expansion resulted in enhanced cytotoxicity against lymphoma cell targets and increased expression of genes related to NK effector pathways when compared to expanded cord-blood NK cell controls. When complexed with a bispecific CD30/CD16 antibody (AFM13), the expanded pre-activated cord-blood NK cells also exhibited enhanced responses against CD30^+^ lymphomas in vitro and in vivo. Based on these results, a clinical trial (NCT#04074746) is underway in patients with relapsed/refractory CD30^+^ lymphomas using cytokine-pre-activated and feeder cell-expanded cord-blood NK cells that were complexed with AFM13 [48].

We have previously shown the use of the extracellular domain of human TF as a protein scaffold for expression of cytokine, ligand, receptor, and single-chain antibody domains [17, 30, 49]. This platform allows high-level mammalian cell expression of difficult-to-produce proteins (i.e., IL-15). Additionally, these TF fusions can comprise single chain polypeptides as well as multi-chain multi-functional complexes, all of which can be readily purified by anti-TF antibody affinity chromatography. This protein expression strategy is scalable using standard commercial manufacturing methods and multiple lots of these fusion proteins have been produced under GMP conditions for use in clinical studies as injectable therapeutics and as ex vivo reagents supporting cell-based therapies. We have also developed methods to balance the biological activities of multi-domain fusion proteins to provide optimal immunostimulatory and/or targeting activities [17, 30]. The development and characterization of HCW9206 comprising IL-7/TF/IL-15 and IL-21/IL-15RαSu polypeptides, described here, represent an extension of these strategies in creating a triple cytokine fusion protein complex to activate and expand human NK cells ex vivo. Interestingly, binding of the anti-TF antibody, HCW9101, to HCW9206 potentiates this effect on NK cells via the CD16 binding activity of the HCW9101 IgG1 domain. This further verifies that the TF fusion protein platform is highly tunable in allowing combinations of different functional domains in a single protein complex and supports its use in designing novel therapeutics capable of stimulating a variety of different biological effects.

In summary, we present an approach to expand human blood NK cells that have been programmed via the IL-12/15/18 receptors to exhibit memory-like properties. This approach overcomes several barriers to GMP NK cell expansion coupled with endowing the expanded NK cells with prolonged in vivo functional persistence. A similar overall cell expansion strategy has been scaled up for production of NK cell clinical products (> 10^11^ cell/lot) that are currently being evaluated in clinical trials in rel/ref AML (NCT#05470140). Further investigation of “Prime and Expand” ML NK cells is warranted to define their relevance for treatment of solid tumors.

## Supplementary Information

Below is the link to the electronic supplementary material.

Supplementary file1 (PDF 412 KB)

## Data Availability

The proprietary materials are available in limited quantities at a reasonable cost to the scientific community for non-commercial purposes, upon timely request under a material transfer agreement through HCW Biologics Inc.
