# Supplementary material for: A “Prime and Expand” strategy using the multifunctional fusion proteins to generate memory-like NK cells for cell therapy
Source: Cancer Immunol Immunother. 2024 Jul 3;73(9):179. doi: 10.1007/s00262-024-03765-8 (PMC11222348; doi:10.1007/s00262-024-03765-8)

Supplementary Table 1. List of Mass Cytometry Antibodies.

Channel	Antigen	Clone	Source	Cat#, RRID
089Y	CD45	HI30	FLUIDIGM	3089003B; RRID:AB_2661851
141Pr	Ki-67	Ki-67	Biolegend	350523; RRID:AB_2562838
142Nd	CD3	UCHT-1	Biolegend	300443; RRID:AB_2562808
142Nd	CD14	M5E2	Biolegend	301843; RRID:AB_2562813
142Nd	CD19	HIB19	FLUIDIGM	3209003B; RRID:AB_2687654
143Nd	KIR3DL1	DX9	RD Systems	MAB1225; RRID:AB_2130828
144Nd	CD38	HIT2	FLUIDIGM	3144014B, RRID:AB_2687640
145Nd	KIR2DS4 (CD158i)	FES172	Beckman	Custom, RRID:NA
146Nd	KIR2DL1/DS1 (CD158a,h)	HP-3E4	BD Biosciences	556061; RRID:AB_396330
147Sm	NKG2D	1D11	BD Biosciences	562164; RRID:AB_10893343
148Nd	KIR2DL2/2DL3 (CD158b)	CH-L	Beckman	IMBULK1B, RRID:NA
149Sm	CD98	UM7F8	BD Biosciences	556074; RRID:AB_396341
150Nd	Mip1a	93342	RD Systems	MAB2701, RRID: AB_2259652
151Eu	TRAIL	RIK-2	Biolegend	308202; RRID:AB_345288
152Sm	CD8	SK1	Biolegend	344702; RRID:AB_1877104
153Eu	CD71	M-A712	BD Biosciences	555534; RRID:AB_395918
154Sm	KIR2DL5 (CD158f)	UP-R1	Miltenyi Biotec	130-096-200; RRID:AB_2660366
155Gd	EOMES	WD1928	Invitrogen	14-4877-82; RRID:AB_2572882
156Gd	PD-L1	29E.2A3	FLUIDIGM	3156026B; RRID:AB_2687855
156Gd	PD-L2	24F.10C12	Biolegend	329602; RRID:AB_1089010
158Gd	CD137	4B4-1	FLUIDIGM	3158013B, RRID:AB_2888927
159Tb	NKG2C	134591	RD Systems	MAB138; RRID:AB_2132982
160Gd	CD69	FN50	Biolegend	310902; RRID:AB_314837
161Dy	NKp30	P30-15	Biolegend	325204; RRID:AB_756108
162Dy	LAG-3	11C3C65	Biolegend	369302, RRID:AB_2616876
163Dy	GLUT-1	202915	RD Systems	MAB1418; RRID:AB_2191039
164Dy	T-bet	4B10	BD Biosciences	561262, RRID: AB_10565981
165Ho	CD16	3G8	FLUIDIGM	3165001B; RRID:AB_2802109
166Er	NKG2A	Z199	BD Biosciences	Custom, RRID:NA
167Er	NKp44	P44-8	Biolegend	325104; RRID:AB_756096
168Er	DNAM-1	DX11	BD Biosciences	559787; RRID:AB_397328
169Tm	CD25	2A3	FLUIDIGM	3169003B; RRID:AB_2661806
170Er	NKp80	239127	Novus	MAB1900; RRID:NA
171Yb	Granzyme B	GB11	FLUIDIGM	3171002B; RRID:AB_2687652
172Yb	CD57	HCD57	FLUIDIGM	3172009B, RRID:AB_2888930
173Yb	TGFbR2	W17055E	Biolegend	399702; RRID:AB_2861013
174Yb	NKp46	9E2	BD Biosciences	557911; RRID:AB_396933
175Lu	Perforin	B-D48	FLUIDIGM	3175004B, RRID:NA
176Yb	CD56	NCAM16.2	FLUIDIGM	3176008B; RRID:AB_2661813
209Bi	CD11b	ICR44	FLUIDIGM	3209003B; RRID:AB_2687654

Supplementary Figure 1. Schematic representation of the human IFNγ gene. The IFNγ coding sequence (Exons 1-4, blue boxes), the proximal promoter ($P_{Ifn\gamma}$, black box), and the distal conserved noncoding sequence 1 enhancer (CNS-1, green box) regions are depicted. DNA sequences of $P_{Ifn\gamma}$ (228 bp), and CNS-1 (247 bp) amplicons are presented with the binding regions for the PCR primers (lower case letters, IFNG127F, IFNG355R-bio, IFNG-CNS1F, and IFNG-CNS1R-bio) and the pyrosequencing primers (underlined uppercase bold letters, C186-IFNG135F, C54-IFNG261F, C4399-CNS1F, and C4293-CNS1F). The CpG sites within the $P_{Ifn\gamma}$ and CNS-1 regions are highlighted in yellow with the four informative CNS-1 methylated CpG sites boxed (location -4360, -4325, -4293, and -4278 relative to the IFNγ transcription start site).

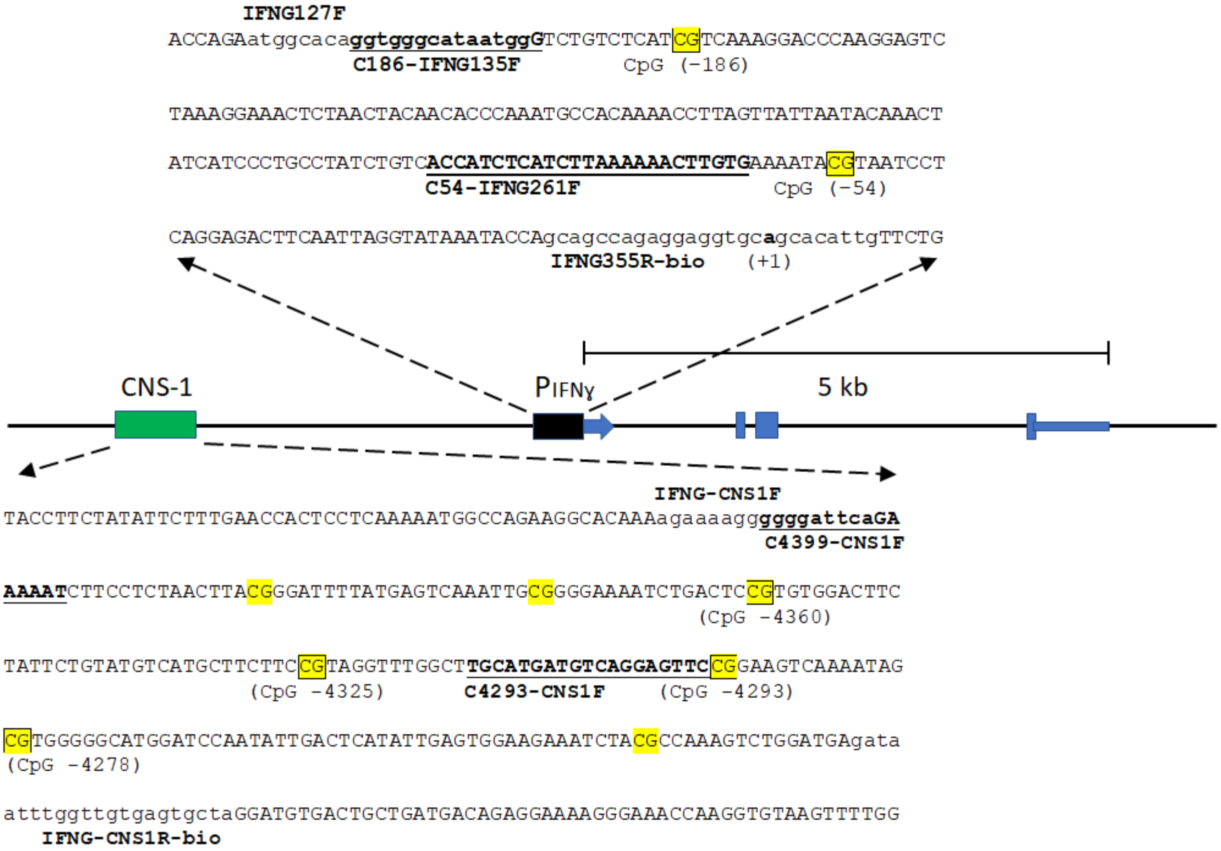

Supplementary Figure 2. *In vivo* antitumor activities of "Expand" and "Prime and Expand" NK cells against THP-1 cells in NSG mouse model.
.

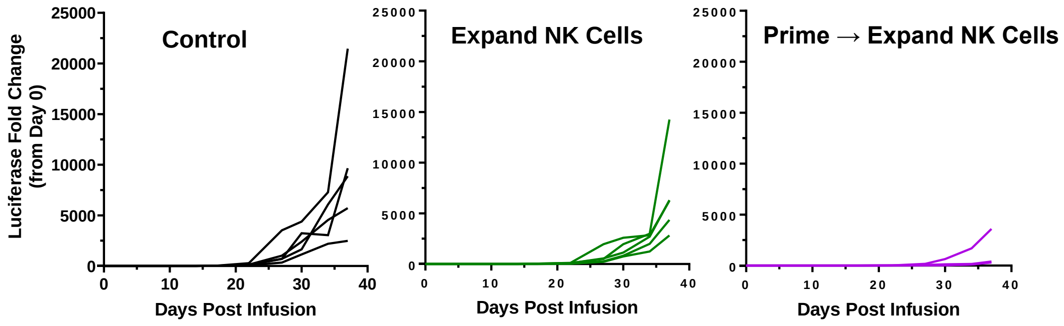

Supplementary Figure 3. *In vivo* antitumor activities of "Expand" and "Prime and Expand" NK cells against THP-1 cells in NSG mouse model. Data from Figure 7C plotted as BLI flux in log-scale to reflect tumor burden and differences in treatment groups at early time points post cell infusion.
.

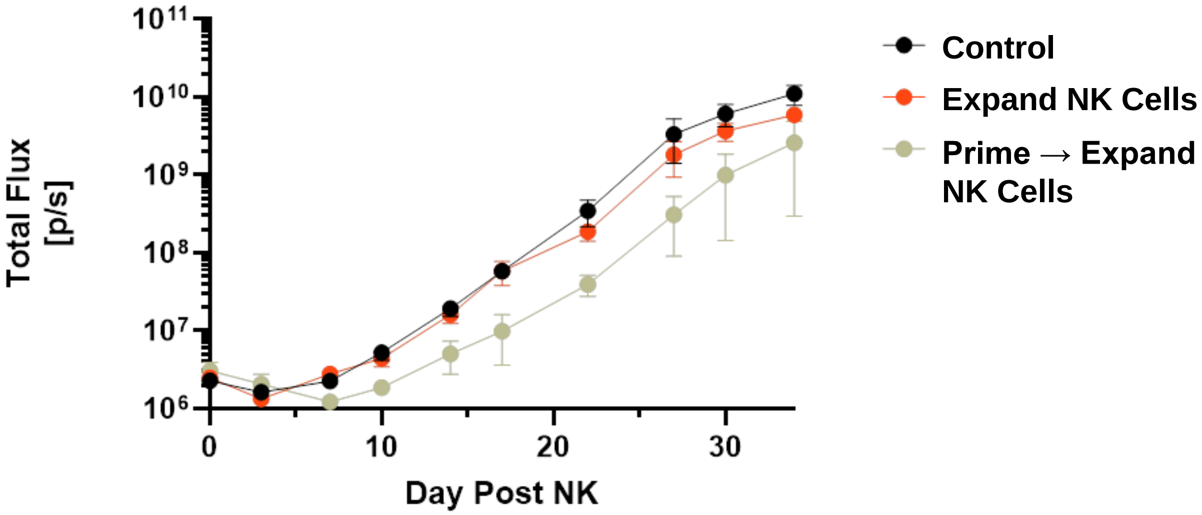

Supplementary Figure 4. Strategy for "Prime and Expand" for feeder-cell free approach for efficient production of clinical-grade ML NK cells.

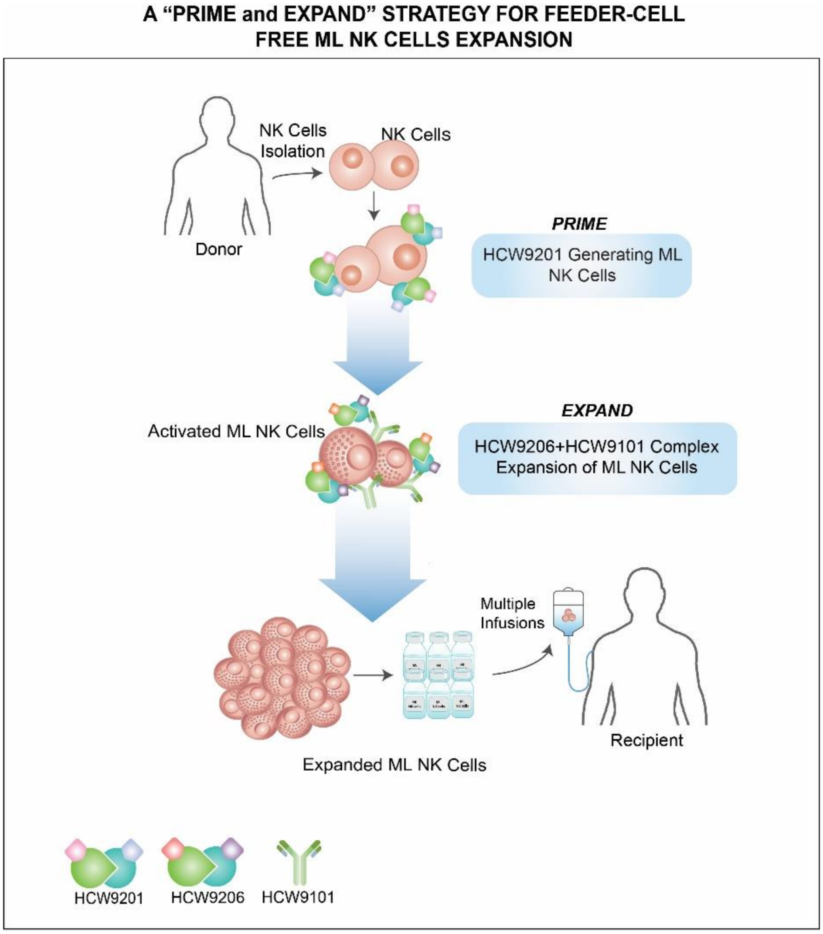

Supplement: Supplementary file 1 — Supplementary file1 (PDF 412 KB) [file 262_2024_3765_MOESM1_ESM.pdf]
